# Carboxylesterase gene amplifications associated with insecticide resistance in *Aedes albopictus*: Geographical distribution and evolutionary origin

**DOI:** 10.1371/journal.pntd.0005533

**Published:** 2017-04-10

**Authors:** Linda Grigoraki, Dimitra Pipini, Pierrick Labbé, Alexandra Chaskopoulou, Mylene Weill, John Vontas

**Affiliations:** 1 Institute of Molecular Biology and Biotechnology, Foundation for Research and Technology-Hellas, Heraklion, Greece; 2 Department of Biology, University of Crete, Heraklion, Greece; 3 Institut des sciences de l’évolution, CNRS–IRD–Université de Montpellier-EPHE, Montpellier, France; 4 European Biological Control Laboratory, USDA-ARS, Thessaloniki, Greece; 5 Department of Crop Science, Pesticide Science Lab, Agricultural University of Athens, Athens, Greece; Fundaçao Oswaldo Cruz, BRAZIL

## Abstract

**Background:**

*Aedes albopictus* is one of the most invasive human disease vectors. Its control has been largely based on insecticides, such as the larvicide temephos. Temephos resistance has been associated with the up-regulation, through gene amplification, of two carboxylesterase (CCE) genes closely linked on the genome, capable of sequestering and metabolizing temephos oxon, the activated form of temephos.

**Principal findings:**

Here, we investigated the occurrence, geographical distribution and origin of the CCE amplicon in *Ae*. *albopictus* populations from several geographical regions worldwide. The haplotypic diversity at the *CCEae3a* locus revealed high polymorphism, while phylogenetic analysis showed an absence of correlation between haplotype similarity and geographic origin. Two types of esterase amplifications were found, in two locations only (Athens and Florida): one, previously described, results in the amplification of both *CCEae3a* and *CCEae6a*; the second is being described for the first time and results in the amplification of *CCEae3a* only. The two amplification events are independent, as confirmed by sequence analysis. All individuals from Athens and Florida carrying the *CCEae3a-CCEae6a* co-amplicon share a common haplotype, indicating a single amplification event, which spread between the two countries.

**Significance:**

The importance of passive transportation of disease vectors, including individuals carrying resistance mechanisms, is discussed in the light of efficient and sustainable vector control strategies.

## Author summary

Control of mosquito borne diseases is being seriously challenged by the ongoing development of insecticide resistance. Resistance of *Aedes albopictus*, a major arbovirus vector, to the organophosphate larvicide temephos was recently associated with the up-regulation, through gene amplification, of two carboxylesterases; *CCEae3a* and *CCEae6a*. Here we investigated the worldwide distribution and origin of the amplified esterases, which is of great value for designing and implementing efficient vector control programs. Individuals with amplification of both esterases were found in Greece and Florida (U.S.A), representing a single amplification event that spread between the two countries, highlighting the importance of passive transportation of disease vectors carrying resistance mechanisms, which is mainly facilitated by human activities. In addition, individuals with amplification of the *CCEae3a* only, but not the *CCEae6a*, representing a second and independent amplification event were found in Florida. The worldwide haplotypic diversity obtained for *CCEae3a* is consistent with the highly invasive nature of the *Aedes albopictus*.

## Introduction

*Aedes albopictus* is vector of several important arboviruses, and has recently become a major threat to human health [1,2]. Its role in several disease outbreaks has been documented, for example dengue in Hawaii (2001–02), Gabon (2007) and Japan (2014)[3–5]; chikungunya in Italy (2007) and Reunion Island (2005–06) [6,7]; Zika in Gabon (2007) [8]. This mosquito species has also drawn attention by being one of the most invasive human disease vectors worldwide. It originated from India and South-East Asia, and quickly invaded almost all continents [9]. Its presence was first reported in Europe (Albania) in 1979 [10], in USA (Texas) in 1985 [11] and in Africa (Cape Town) in 1989 [12]. The successful spread of *Ae*. *albopictus* throughout the globe has been facilitated by human activities, like trade of tires and other goods, as well as tourism [2]; these indeed allow transportation of desiccated eggs and larvae to new places. Its ability to adapt to different environments has also been associated with specific biological traits, such as the production of diapausing eggs, which enables its survival at cooler temperatures [9].

The control of *Ae*. *albopictus* is largely based on habitat management campaigns [13], repellents (spatial or personal) and insecticides (larvicides and adulticides) [14], while the use of alternative means such as *Wolbachia*, Sterile Insect Techniques and genetic manipulation approaches are also currently being investigated [15,16]. Among a limited number of mosquito larvicides (including bacterial toxins and insect growth regulators—IGRs), temephos is an organophosphate (OP) that has been used extensively for the control of *Aedes* mosquitoes (*Ae*. *aegypti* and the often sympatric *Ae*. *albopictus*) in several continents and countries [17]. However, resistance against this insecticide has been reported [18,19]. We recently showed [20] that resistance against temephos in an *Ae*. *albopictus* population from Greece is associated with the upregulation, through gene amplification (*i*.*e*. multiple gene copies) of two carboxylesterase genes (CCEs), namely *CCEae3a* and *CCEae6a*, which are closely located on the genome [21]. Notably, the same orthologous genes (*CCEae3a* and *CCEae6a*) have also been associated with temephos resistance in *Ae*. *aegypti*, the primary dengue and yellow fever vector worldwide [22]. CCEae3a protein was shown to be localized in malpighian tubules (MT) and nerve tissues of the *Ae*. *albopictus* larvae, as well as being able to sequester and metabolize temephos oxon, the activated form of temephos [23].

OP resistance based on sequestration and enhanced metabolism resulting from CCE gene amplifications has also been described in other insects and mosquito species, such as the aphid *Myzus persicae* [24] and members of the mosquito *Culex pipiens* complex [25,26]. At the world scale, only a few CCE genes have been recorded amplified in cases of resistance in insects, which tends to indicate that advantageous mutations could be limiting. Although the mechanism by which esterase genes are amplified has not been established yet, it has been suggested that certain genome regions are probably a "hot spot" for recombination and amplification. Regions showing homology with repetitive elements have been found in DNA flanking the amplified CCEs, suggesting that these may have a role in the amplification process, as they may be functionally related to transposable elements [27].

Resistance to OPs in *Culex* mosquitoes has been shown to occur via over-expression, through gene amplification, of two esterase loci, *Est-2* and *Est-3*, which may be amplified singly (e.g. the estβ1 gene) or more commonly are co-amplified as allelic pairs in resistant mosquitoes [27,28]. However, the amplified alleles can differ, which indicates that the amplification process happened several times independently. These amplified CCE alleles have been described in different geographical places. Some of them remained localized in a relatively limited area and appeared as independent events. Others have spread to distant regions from a single evolutionary origin; the same common haplotypes are indeed found in mosquitoes from different continents [29–32]. It appears that once amplification has occurred, it can easily reach other geographic areas by migration, and then invade thanks to local insecticide selection [33]. For example, the worldwide most common allele is *Ester*^*2*^ (or estα2-estβ2 co-amplicon), which occurs in >80% of insecticide resistant strains [34], suggesting that it may confer higher fitness than other allelic variants [33,35].

The distribution and origin of amplified CCEs associated with insecticide resistance has not been studied in *Aedes* mosquitoes. Here, we investigated the occurrence, frequency and geographical distribution, as well as the phylogenetic relationship and origin of the *CCEae3a*-*CCEae6a* amplicon(s)/loci in *Ae*. *albopictus* populations from 16 different places across the globe.

## Materials and methods

### Sampling and species ID verification

*Ae*. *albopictus* field mosquitoes used in this study were collected from Mexico (Apocada, Reynosa and Tapachula), U.S.A (Florida and Atlanta), Brazil (Rio de Janeiro), Belize (Orange walk town), Gabon (Franceville, Cocobeach, Lope), Switzerland (Ticino), France (Montpellier), Italy (Lombardy), Greece (Agios Stefanos, Koronida), Taiwan (Taipei), China (Beijing), Sri Lanka (Peradeniya), Australia (Hammond), Bangladesh (Panchagarh), Lebanon (Beirut) and Japan (Tokyo). In addition individuals from two laboratory colonies were used: i) the Tem-GR strain, derived from an *Ae*. *albopictus* population collected in 2010 in Athens (Greece) and selected with temephos using standard WHO larval bioassays [20], and ii) the Malaysia-Lab strain, a susceptible laboratory strain originally collected in Malaysia [36].

*Ae*. *albopictus* adults or larvae stored in ethanol were first dried, and then genomic DNA was extracted from each individual using the Cethyl Trymethil Ammonium Bromide (CTAB) method described in Navajas et al. [37]. The DNA pellet was dissolved in 20μl of sterile water. Individuals were identified to species based on a species ID PCR [38]. In each PCR reaction, reference *Ae*. *albopictus* and *Ae*.*aegypti* samples were used as controls.

### Detection of esterase gene amplification

*CCEae3a* and *CCEae6a* gene copy number variation (CNV) was assessed using quantitative PCR (qPCR) on individual *Ae*. *albopictus* specimens (S1 Table). Amplification reactions (25μl final volume) were performed on a MiniOpticon Two-Color Real-Time PCR Detection System (BioRad) using 2μl of genomic DNA (diluted 5 times), 0.4μM primers (two different primer pairs per target gene) (S2 Table) and Kapa SYBR FAST qPCR Master Mix (Kapa-Biosystems). Two housekeeping genes, histone3 (NCBI: XM_019696438.1) and the ribosomal protein L34 (NCBI: XM_019677758.1), were used as reference genes for normalization[39]. Fivefold dilution series of pooled genomic DNA from the temephos susceptible Malaysia-Lab strain and the temephos selected TemGR strain were used to assess the efficiency of the qPCR reaction for each gene specific primer pair. A no-template control (NTC) was included to detect possible contamination and a melting curve analysis was performed to check the presence of a unique PCR product. Differences in *CCEae3a* and *CCEae6a* gene copy numbers were estimated relative to the temephos susceptible Malaysia-Lab strain, following Pfaffl [40].

### Sequencing of *CCEae3a* intronic regions

*CCEae3a* (Vector base, AALF007796) is predicted to encompass three exons and two introns. To identify the most variable part of the gene the full intron1 was amplified using forward primer 5’-ACGGTCCTCGATACATAGTG-3’ and reverse primer 5’-TAGCCTCATTGCTGGTTAGC-3’ (hybridizing respectively at the end of exon1 and at the beginning of exon2) and the full intron2 was amplified using forward primer 5’-AGAGTGCGTTACGGATCAAG-3’ and reverse primer 5’-CACTGGCTTCCAGGAGATAC-3’ (hybridizing respectively at the end of exon2 and at the beginning of exon3). The PCR reactions (25μl final volume) were performed using 2μl genomic DNA from individual *Ae*. *albopictus* mosquitoes, 0.4μM primers, 0.2mM dNTPs, 5μl of 10X buffer and 1U of Kapa Taq DNA Polymerase (KAPABIOSYSTEMS). The PCR conditions were 95°C for 5min followed by 29 cycles of 94°C for 30sec, 48°C for 30sec, 72°C for 1min and a final extension of 72°C for 10min. PCR products were purified using a PCR purification kit (Macherey Nagel) and sent for sequencing using the forward primer (Macrogen Sequencing Facility, Amsterdam).

To assess the diversity of *CCEae3a*, the 709bp fragment of the gene (including the last 314bp of exon1, the whole intron1 and the first 192bp of exon2) was sequenced. PCR products from homozygous individuals were sequenced directly using the forward primer (5’-ACGGTCCTCGATACATAGTG-3’); for heterozygotes, the PCR products were cloned using the pGEM-Teasy vector (Promega) according to manufacturer’s instructions to separate the different alleles, and six clones for each individual were sent for sequencing (Macrogen sequencing facility, Amsterdam), with the T7 universal primer. Sequences were examined and aligned using the BioEdit software.

### Phylogenetic tree construction

Phylogenetic relationships between the different *CCEae3a* haplotype sequences were determined using the Phylogeny.fr platform (‘‘one click mode”) [41]. Briefly, sequences were aligned using the MUSCLE 3.8.31 algorithm, and alignment was then refined using the Gblocks 0.91b software to exclude poorly aligned parts. Subsequently the PhyML 3.1/3.0 (aRLT) software was used to assess the clade support, by computing the maximum likelihood tree and aLRT test (approximate Likelihood Ratio Test) [42]. Finally the tree was drawn using the TreeDyn 198.3 software [43].

### Ethics statement

The work described in this manuscript is in no way linked (directly or indirectly) to ethical concerns. No data from humans have been collected. All research activities respect fundamental ethics principles, including those reflected in the Charter of Fundamental Rights of the European Union (2000/C 364/01). The work is compatible with EU and international law, as a number of entomological monitoring activities (and transport of gDNA in Ethanol) is contacted worldwide and in Europe (European Mosquito Control Association, http://www.emca-online.eu).

## Results

### Geographic distribution of *CCEae3a* and *CCEae6a* gene amplification

**Fig 1:**
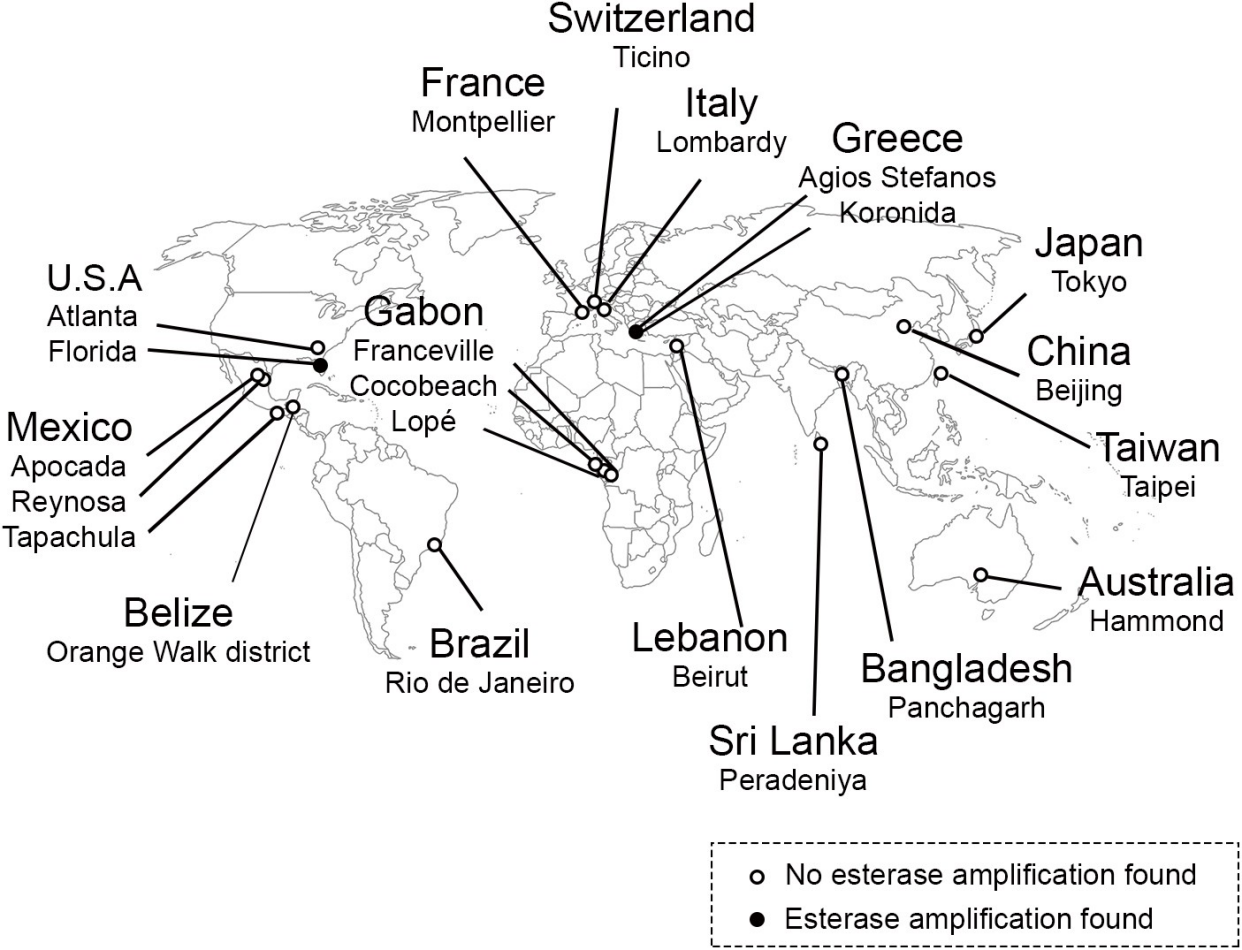
Sample collection map. **The countries of origin for the 385 individuals screened for *CCEae3a* and *CCEae6a* gene amplification are indicated**. Black and white circles respectively represent places were amplification was or not detected.

**Fig 2:**
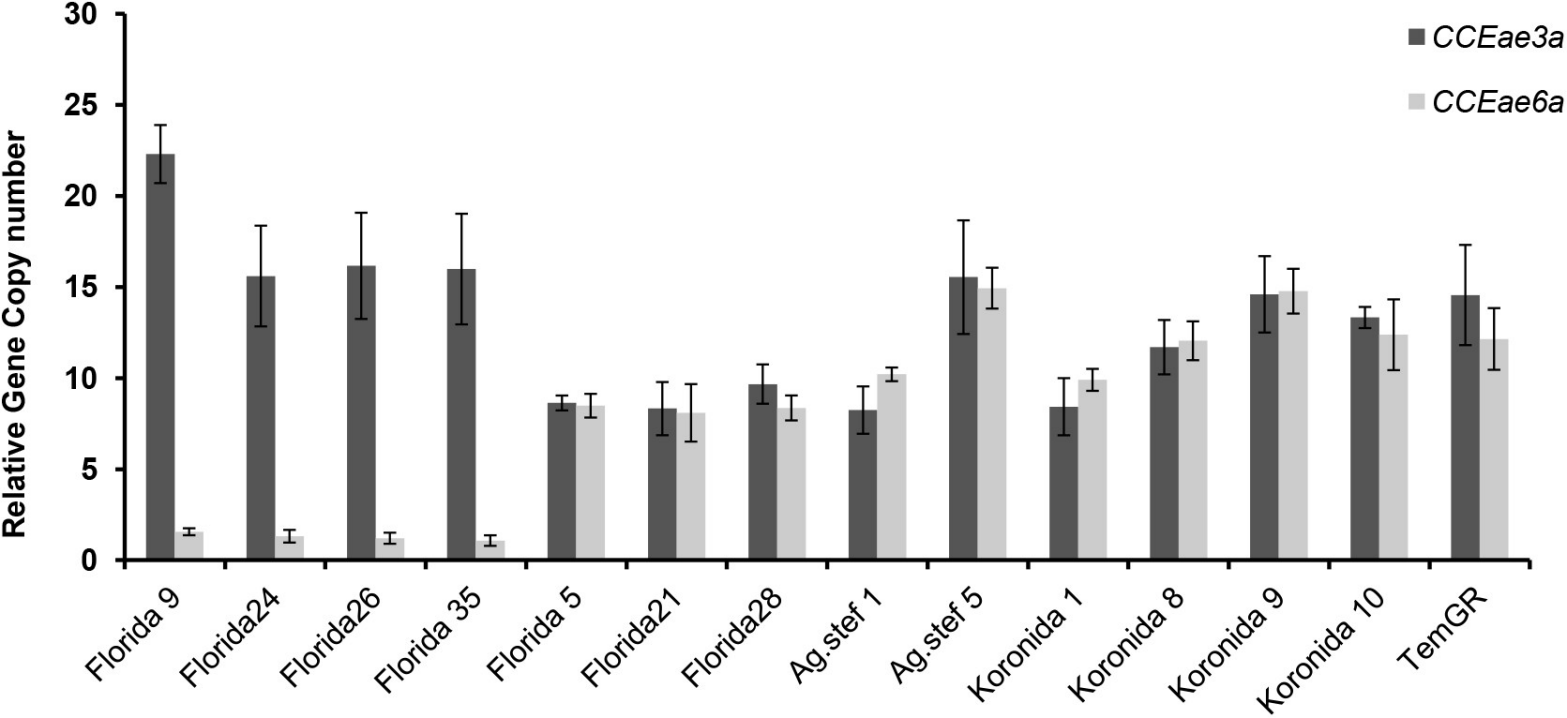
*CCEae3a* and *CCEae6a* gene copy number variation in individuals from Florida and Greece. Quantitative real time PCR values are represented relatively to the values of reference individuals from the Lab strain. Error bars represent the standard error of the mean from at least three technical replicates. *Histone 3* and *rpl34* have been used as reference genes.

A total of 385 mosquitoes from 16 countries and 22 different collection sites (Fig 1 and S1 Table) were first confirmed to be *Ae*. *albopictus* (species ID PCR) and subsequently tested for *CCEae3a* and *CCEae6a* CNV via qPCR. Out of 35 individuals tested from Florida, three showed amplification of both *CCEae3a* and *CCEae6a* (Florida 5, 21 and 28), while four showed amplification of *CCEae3a* only (Florida 9, 24, 26 and 35) (Fig 2). Amplification of both esterases was also detected in Greece: two individuals out of 10 from Agios Stefanos (Ag.stef 1, 2), and four out of 10 from Koronida (Koronida 1, 8, 9, 10). Amplification of both esterases was also confirmed in all tested individuals of the temephos resistant TemGR strain (Fig 2). None of the 330 individuals tested from the remaining 14 countries showed amplification of *CCEae3*a or *CCEae6a* (S1 File).

### Phylogenetic relationships of *CCE3ae* locus/amplicon: At least two independent amplification events have occurred

**Fig 3:**
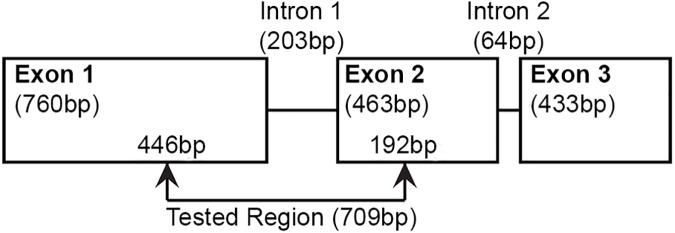
*CCEae3a* gene structure. *CCEae3a* consists of three exons and two introns, represented by boxes and lines respectively, with their size in base pairs (bp). The region selected for the phylogenetic analysis is indicated by the arrows.

The two predicted introns of the *CCEae3a* locus were sequenced using individuals from the TemGR and Malaysia-Lab strains. Intron1 sequences were longer and more variable between individuals from these two strains, while intron2 sequences were identical. A 709 bp region including part of exon1, the whole intron1 and part of exon2 (Fig 3) was thus used to examine the haplotype diversity between individuals from the sampled countries, with and without CCE amplification (1–14 individuals per collection site, S1 Table).

Sequence alignment revealed several SNPs throughout the amplified region, both in introns and exons, plus some insertions and deletions in the intronic sequence (S2 File). The diversity was higher in the intron sequences (mean distance = 0.041 substitution/site) than in the exons (0.012 and 0.014 for exon1 and exon2, respectively)[44]. A total of 45 different haplotypes, differing by at least one mutation, were identified from the 49 individuals tested (S2 File).

**Fig 4:**
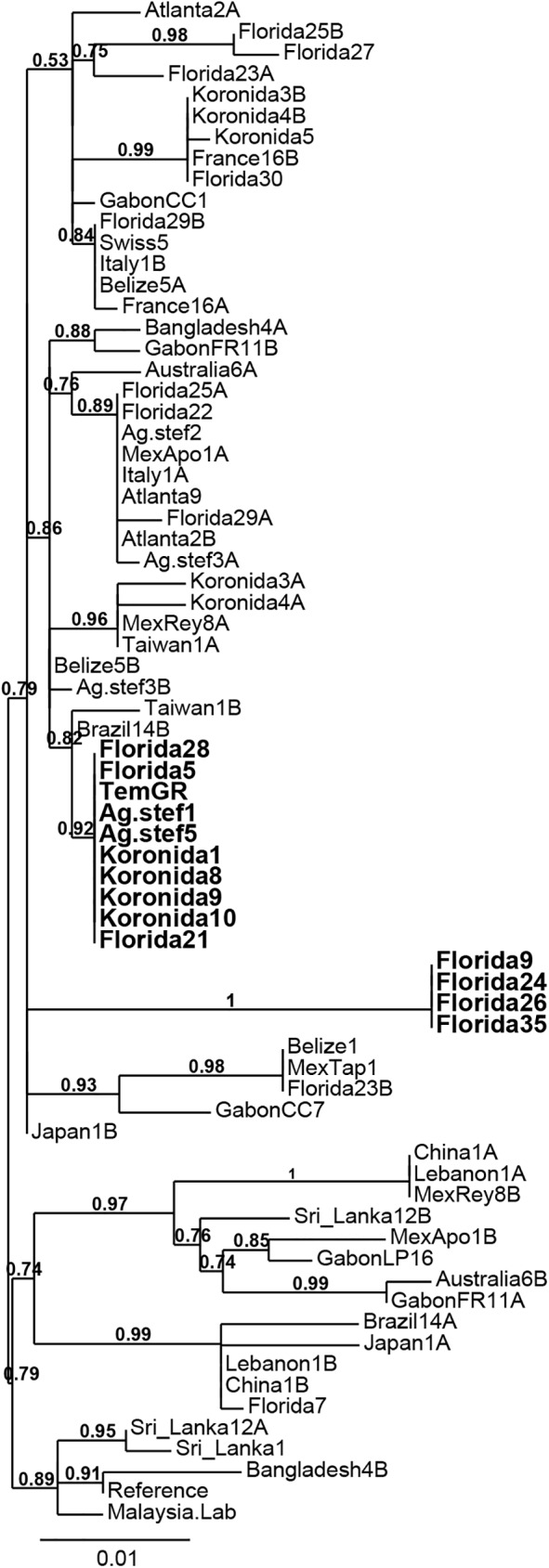
*CCEae3a* diversity. An unrooted maximum likelihood tree was built to represent the phylogenetic distances between the 45 *CCEae3a* haplotypes identified worldwide (see also S1 Table). Distances are expressed in substitutions/site. The numbers on tree branches represent the supporting probability of each node, based on the aLRT statistical test. Individuals with copy number variation are shown with bold.

Haplotypes did not cluster based on geographic proximity. While most haplotypes were found in a single individual, some sequences from individuals collected in distant geographic locations were indeed identical: for example H3 was present in individuals from Atlanta, Italy, Greece (Agios Stefanos), Florida and Mexico (Apocada), H7 in individuals from Belize, Italy, Florida and Switzerland, H9 in individuals from Belize, Florida and Mexico (Tapachula), H12 in individuals from China and Lebanon, and H13 in individuals from China, Lebanon and Mexico (Reynosa). Moreover, sequences obtained from individuals collected in the same area often showed a great variability and were found in different clades in the tree, to the point that two sequences found in a single heterozygous individual could be quite distant (e.g. Bangladesh4A and B or Brazil14A and B). In particular, haplotypes obtained from individuals with no esterase amplification originating from Florida and Greece were dispersed throughout the phylogenetic tree (Fig 4), clustering with haplotypes from distant areas. In contrast, all individuals displaying amplified esterases clustered in only two highly supported clades. In the first, all the individuals from Florida (U.S.A) showing amplification of only *CCEae3a* shared a common haplotype (H29), which was found in no other individual in the dataset. The second clade clustered all the individuals showing amplification of both *CCEae3a* and *CCEae6a*, whether from Florida (U.S.A) or from Greece (Agios Stephanos and Koronida), including the reference strain TemGR. They also shared a common haplotype (H30), which again was found in no other individual in the dataset. In addition, this second clade was closer to haplotypes obtained from individuals without esterase amplification (e.g Brazil 14B and Taiwan 1B) than to the first clade (*i*.*e*. individuals with amplification of *CCEae3a* only).

## Discussion

Two carboxylesterase genes (CCEs), *CCEae3a* and *CCEae6a* have recently been shown in *Ae*. *albopictus* to be implicated in OP resistance through gene amplification [20]. To understand the origin and spread of these resistance alleles, we assessed the haplotypic diversity at the *CCEae3a* locus. Analysis revealed that this gene is polymorphic in non-amplified alleles (S2 File): 45 different haplotypes were identified in 49 individuals collected around the globe, with only five (H3, H7, H9, H12 and H13) shared between two or more individuals. Moreover, for these five haplotypes, the individuals came from distant areas, on different continents. The phylogenetic tree further confirmed the absence of correlation between haplotype similarity and geographic origin, as individuals from the same collection area clustered mostly in different clades of the tree (this was even observed for the two haplotypes of a single heterozygote). This observation echoes previous studies on mitochondrial genes, microsatellites and other nuclear genetic markers [45–47] that showed that *Ae*. *albopictus* populations have been repeatedly transported from their original range (South-East Asia) to different areas around the globe; progenies of mosquitoes originally from the same locality can thus be found in different continents. The frequent exchange of goods at an international level and travelling of people around the world indeed facilitate the passive transportation of mosquitoes, which are often found in aircrafts and ships [48]. These studies also showed that the non-Asiatic populations often result from a mix of several independent invasions. This mechanism, promoting genetic diversity, is often proposed as a key factor contributing to successful establishment of a species in new areas [45,49].

We then addressed the worldwide occurrence, distribution and diversity of esterase amplification in *Ae*. *albopictus* populations in response to OP selection: our study evidenced that at least two types of esterase amplifications are currently segregating. The first one, resulting in amplification of both *CCEae3a* and *CCEae6a*, had been previously described [20], while the second; resulting in amplification of *CCEae3a* only, is being described for the first time. In contrast to the high *CCEae3a* haplotypic diversity in non-amplified alleles, the two amplified genotypes associated with OP resistance displayed no diversity. It also revealed that the two amplification events were independent: individuals with *CCEae3a* and *CCEae6a* co-amplification displayed identical sequences and thus clustered together, far apart from individuals with *CCEae3a*-only amplification, which also displayed identical sequences. The appearance of independent amplification events at the same genomic region suggests the presence of favoring features, which promote unequal crossing-overs and/or transposition [27]. For example, a repetitive element Juan (possibly related to transposable elements) was found close to the amplified esterase locus in *Cx*. *quinquefasciatus* [50]. The *Ae*. *albopictus* genome is also known to carry many transposable elements, and 68% of its genome is occupied by repetitive sequences [21]. If one of those is close to the *CCEae3a* locus, it could facilitate its repeated and independent duplications. In addition, these mechanisms might also act after the first amplification event resulting in further variation in copy numbers. This has been hypothesized in *Cx*.*pipiens* [51] and might also explain the differences observed in the relative gene copy numbers among individuals from Florida and Greece. The fact that all individuals carrying co-amplifications of both *CCEae3a* and *CCEae6a*, from Greece and Florida U.S.A (two regions hosting many millions of tourists every year), share a common haplotype reveals that a single amplification event took place, and then spread between the two countries. This again outlines the importance of passive transportation of disease vectors, including individuals carrying resistance mechanisms, which unfortunately promotes resistance spread at the world scale, as has been described in *Cx*. *pipiens* mosquitoes with OP-resistant amplified esterases [30,52]. However, the establishment of a resistance mechanism in a new area largely depends on the local selective advantage it offers in these new environmental conditions [33]. The repetitive use of temephos [53] or other organophosphate insecticides, which could show cross resistance, like the adulticide naled commonly used in Florida [54] probably facilitated the establishment of *CCEae3a* amplified haplotypes in *Ae*. *albopictus* populations from both countries. It also ensured that these haplotypes were selected and reached high frequencies.

It is actually surprising that the resistance allele found in individuals from the TemGR strain, originally collected in 2010, is still present in Greece 2016 collection: temephos has been officially banned in Europe in 2007, and resistance mechanisms are usually (but not always) associated with fitness costs [55,56]. The persistence of the resistant allele throughout these years suggests either the lack of a significant fitness cost for individuals carrying them or the presence of a current selection source, either from non officially approved vector control activities, or from other substances (e.g. originating for example from agriculture) [57].

In any case, the presence of two independently amplified OP-resistant alleles in *Ae*. *albopictus* already segregating in distant places in the world (*i*.*e*. Athens—Greece and Florida–U.S.A) raises concerns about the future control of this species. Although the levels of insecticide resistance in *Ae*. *albopictus* are low at present (*i*.*e*. resistance mechanisms/alleles are less frequent) compared to *Ae*. *aegypti* [18,58], our study shows that resistance can be selected and spread rapidly around the globe also in this species and possibly compromise control activities. As only a limited number of mosquito larvicides are available on the market, temephos resistance is an important consideration for many countries, where this active ingredient is still in use. Moreover, striking resistance mutations have been found in other insect species (*Plutella xylostella*) that completely inactivate the IGR diflubenzuron, now one of the most important mosquito larvicides in Europe and other regions [59]. This further raises serious concerns for regions that have banned the use of temephos, such as Europe, as it is certainly a potential reliable resource for emergency epidemics, new invasion cases, or lack of alternative efficient mosquito control solutions.

## Supporting information

S1 Table Country-location, number of *Aedes albopictus* individuals used in the study and haplotypes identified. (DOCX)

S2 Table Primers used for quantitative real time PCR. (XLSX)

S1 File qPCR results for gene copy number variation of CCEs in all tested individuals. (XLSX)

S2 File All variable sites in the amplified part of CCEae3a esterase. (XLSX)
