# Supplementary material for: Carboxylesterase gene amplifications associated with insecticide resistance in Aedes albopictus: Geographical distribution and evolutionary origin
Source: PLoS Negl Trop Dis. 2017 Apr 10;11(4):e0005533. doi: 10.1371/journal.pntd.0005533 (PMC5398709; doi:10.1371/journal.pntd.0005533)
Supplement: S1 Table — (DOCX) [file pntd.0005533.s001.docx]

**S1 Table: Country-location, number of *Aedes albopictus* individuals used in the study and haplotypes identified.**

| **Country-Locality** | **Year of collection** | **N_ad_^1^** | **N_s_^2^** | **Haplotypes^3^** |
| --- | --- | --- | --- | --- |
| Australia | 02-04/2012 | 20 | 1 | Australia6_A(H1), Australia6_B(H2) |
| U.S.A- Atlanta | 08/2015 | 31 | 2 | Atlanta2_A(H4), Atlanta2_B(H3), Atlanta9(H3) |
| Bangladesh | 08/2015 | 9 | 1 | Bangladesh4_A(H5), Bangladesh4_B(H6) |
| Belize |  | 20 | 2 | Belize5_A(H7), Belize5_B(H8), Belize1(H9) |
| Brazil | 07/2015 | 20 | 1 | Brazil14_A(H10), Brazil14_B(H11) |
| China | 06-07/2015 | 25 | 1 | China1_A(H13), China1_B(H12) |
| Gabon-Cocobeach | 06-07/2015 | 15 | 2 | GabonCC1(H14), GabonCC7(H15) |
| Gabon-Franceville | 06-07/2015 | 16 | 1 | GabonFR11_A(H16), GabonFR11_B(H17) |
| Gabon-Lope | 06-07/2015 | 15 | 1 | Gabon LP16(H18) |
| Italy | 07-11/2012  05-11/2013 | 20 | 1 | Italy1_A(H3), Italy1_B(H7) |
| Lebanon | 10/2011 | 10 | 1 | Lebanon1_A(H13), Lebanon1_B(H12) |
| Mexico-Apocada | 04-05/2015 | 10 | 1 | MexApo1_A(H3), MexApo1_B(H19) |
| Mexico-Reynosa | 04-05/2015 | 10 | 1 | MexRey8_A(H20), MexRey8_B(H13) |
| Mexico-Tapachula | 04-05/2015 | 10 | 1 | MexTapa1(H9) |
| France | 07/2015 | 20 | 1 | Montpellier16_A(H21), Montpellier16_B(H22) |
| Sri Lanka | 10-11/2014 | 19 | 2 | SriLanka12_A(H23), SriLanka12_B(H24), SriLanka1(H25) |
| Taiwan | 11/2014 | 20 | 1 | Taiwan1_A(H26), Taiwan1_B(H27) |
| U.S.A- Florida | 09-10/2014 | 35 | 14 | Florida7(H28), Florida5(H30), Florida21(H30), Florida28(H30), Florida9(H29), Florida24(H29), Florida26(H29), Florida35(H29), Florida22(H3), Florida23_A(H41), Florida23_B(H9), Florida25_A(H3), Florida25_B(H42), Florida27(H43), Florida29_A(H44), Florida29_B(H7), Florida30(H45) |
| Lab Malaysia |  | Used as reference | 1 | Malay.Lab(H31) |
| Greece-Agios Stefanos | 07/2016 | 10 | 4 | Ag.stef1(H30), Ag.stef5(H30), Ag.stef2(H3), Ag.stef3_A(H34), Ag.stef3_B(H35) |
| Greece-Koronida | 07/2016 | 10 | 7 | Koronida1(H30), Koronida8(H30), Koronida9(H30), Koronida10(H30), Koronida3_A(H36), Koronida3_B(H37), Koronida4_A(H38), Koronida4_B(H39), Koronida5(H40) |
| Greece-TemGR | 07/2010 | 20 | 1 | TemGR(H30) |
| Japan | 06-07/2015 | 20 | 1 | Japan1_A(H32), Japan1_B(H33) |
| Switzerland | 07-11/2012  05-11/2013 | 20 | 1 | Swiss5(H7) |

1. Total number of individuals tested for CCE amplification
2. Number of individuals sequenced to assess allele diversity
3. Haplotypes identified: a number next to the country name refers to the specific individual used and an A or B distinguishes between the different haplotypes identified in the same individual (ex. Australia6_A and Australia6_B were found in individual 6 from the Australian sample collection)
